# Integrated Development Environment for EEG-Driven Cognitive-Neuropsychological Research

**DOI:** 10.1109/JTEHM.2020.2989768

**Published:** 2020-05-06

**Authors:** Shoham Jacobsen, Oded Meiron, David Yoel Salomon, Nir Kraizler, Hagai Factor, Efraim Jaul, Elishai Ezra Tsur

**Affiliations:** 1 Department of Computer ScienceJerusalem College of Technology 42730 Jerusalem91160Israel; 2 Clinical Research Center for Brain SciencesHerzog Medical CenterJerusalem91120Israel; 3 Geriatric Skilled Nursing DepartmentHerzog Medical CenterJerusalem91120Israel; 4 Neuro-Biomorphic Engineering Laboratory (NBEL)Department of Mathematics and Computer ScienceThe Open University of Israel 42715 Ra’anana4353701Israel

**Keywords:** Emotiv, Python, working memory in elderly

## Abstract

**Background:** EEG-driven research is paramount in cognitive-neuropsychological studies, as it provides a non-invasive window to the underlying neural mechanisms of cognition and behavior. A myriad collection of software and hardware frameworks has been developed to alleviate some of the technical barriers involved in EEG-driven research. **Methods:** we propose an integrated development environment which encompasses the entire technical “data-collection pipeline” of cognitive-neuropsychological research, including experiment design, data acquisition, data exploration and analysis in a state-of-the-art user interface. Our framework is based on a unique integration between a python-based web framework, time-oriented databases and object-based data schemes. **Results:** we demonstrated our framework with the recording and analysis of an n-Back task completed by 15 elderly (ages 50 to 80) participants. This case study demonstrates the highly utilized nature of our integrated framework with a challenging target population. Furthermore, our results may provide new insights into the correlation between brain activity and working memory performance in elderly people, who are prone to experience accelerated decline in executive prefrontal cortex functioning. **Conclusion:** our framework extends the range of EEG-driven experimental methods for assessing cognition available for cognitive-neuroscientists, allowing them to concentrate on the creative part of their work instead of technical aspects.

## Graphical abstract

We propose an integrated development environment which encompasses the entire technical "data-collection pipeline" of cognitive-neuropsychological research, including experiment design, data acquisition, data exploration and analysis in a state-of-the-art user interface. In human subjects testing, our framework extends the range of EEG-driven experimental methods for assessing cognition available for cognitiveneuroscientists, allowing them to concentrate on the creative part of their work instead of technical aspects.

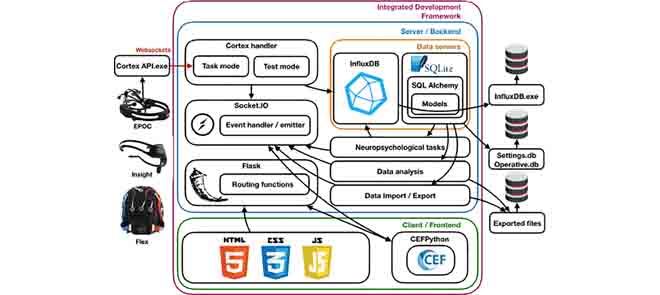

## I. Introduction

Electroencephalogram (EEG) recordings of superimposed neuronal electrical activity can be used to provide insights into the cooperative behavior of neurons, which underlie cognition [1]. As it allows to study the connection between the physiology and the mind, EEG is considered a ‘window on the mind’ [2]. EEG was and is still widely utilized in a broad spectrum of applications ranging from neuromarketing [3] and brain-computer interfaces [4] to clinical and psychological studies [5]. Moreover, it is also utilized to modulate behavior and cognition in neurofeedback studies [6]. In clinical psychology settings, EEG is used to study brain processes underlying attention [7], learning [8], and memory [9], as well as their impairments [10].

EEG is widely utilized across cognitive-neuropsychological research, where a participant is often asked to complete computerized interactive stimuli-driven tasks (requiring participants to respond to certain external sensory stimuli), while his brain activity and task-related responses are recorded. These neuropsychological tasks are usually programmed in a dedicated software suite, such as the commercialized E-Prime [11], the open-sourced PsyToolkit [12], or OpenSesame [13]. Researchers have to ensure proper synchronization of the computerized task with EEG recording by either using an extension product to their E-Prime suite, or by programmatically incorporating the recording into their computerized task. This, by sending triggers to mark the timestamp for significant events (e.g., the onset of a trial, presentation of a particular stimulus, etc.) via a dedicated port to the EEG apparatus. Several frameworks were developed for EEG data analysis including SignalPlant [14], EEGNET [15], NIT [16], or the MATLAB-based toolkit FieldTrip [17] - each has its own pros and cons. This research pipeline of task creation, data acquisition, synchronization and analysis are either expensive or involves significant technical expertise.

Over the past few decades EEG technology has evolved from addressing clinicians needs with cumbersome setups which require long assembly time, hair removal and a wired computer, to small, easily assembled, wireless systems, which can be commercialized for meditation or gaming [18]. Due to the high level of subject cooperation and the essential lengthy clinical-EEG procedures which are required in EEG-driven clinical research, medical-grade EEGs are hard to implement in a variety of clinical or pre-clinical settings, particularly in elderly pre-dementia patients, and hospitalized elderly patients. Therefore, several studies evaluated the success of utilizing modified / non-modified commercialized EEGs in clinical settings [19], [20].

**FIGURE 1.**
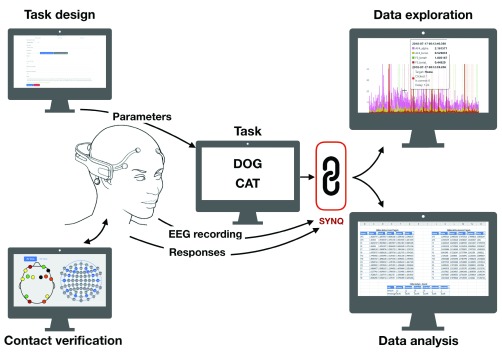
Workflow schematics. Our integrated development environment facilitates task creation, EEG recording, data synchronization and data analysis. It is based on a graphical user interface and does not require programming expertise. The framework currently supports three key types of neuropsychological tasks: N-back task, open vs close eyes task and affective/emotional processing task using the IAPS image repository. The framework currently supports the Emotiv EEG line of products.

Here, we describe a new Integrated Development Environment (IDE), which facilitates task creation, EEG recording, data synchronization and data analysis. Our IDE is based on a Graphical User Interface (GUI) and does not require programming expertise (**Figure 1**). The framework currently supports three key types of neuropsychological tasks and the Emotiv (San Francisco, U.S.A.) EEG line of products. This open source IDE can be easily extended to support other tasks and equipment. We demonstrated the framework by assessing WM performance, using the $n$-Back task [21], in a pre-clinical setting on 15 elderly participants.

## II. Architecture and Implementation

**FIGURE 2.**
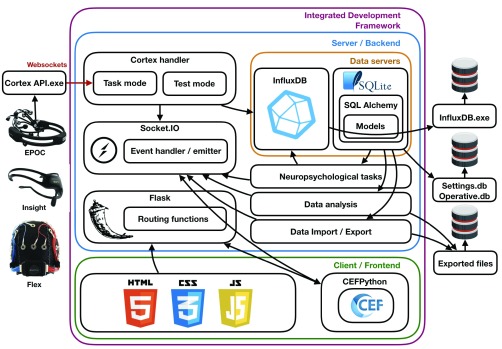
Framework schematics. Framework is based on a server-client model, where the server listens to HTTP-based client’s request and return the corresponding webpage, while utilizing the graphical capabilities of the web browser. The IDE’s backend was based on Python and the front-end on HTML, CSS and Javascript via a web framework. Web frameworks is based on Flask, extended with the Bootstrap and Socket-IO libraries. For user interface design we used the Chromium Embedded Framework (CEF) via the CEFPython package, extended with the pywin32 package. We used two type of databases: SQLite via the sqlite3 package and InfluxDB (Time Series Database) via the influxdb-python package. We used the SQLAlchemy, which we embedded in our Flask-based front-end using the Flask-SQLAlchemy package to define an object-based database architecture. We utilized the ‘Cortex API’ to access Emotiv EEG product line. Data visualization was implemented with the Plotly.js and statistical analysis is provided using python’s numerical analysis package NumPy. Data export was implemented using the xlsxwriter package. Pyinstaller package was utilized to provide an executable compiled environment.

Our proposed IDE facilitates task creation, EEG recording, data synchronization and data analysis and it is based on a server-client model – the server listens to HTTP-based client’s request and return the corresponding webpage. Framework schematics is illustrated in **Figure 2**. Our framework utilizes the advanced graphical capabilities of the web browser to provide a powerful and elegant user interface. In our current IDE, both the server (application) and the client (user) are on the same computer and are therefore communicating via the ‘local host’ IP address. The IDE’s back-end (server side) was based on Python – one of the most utilized environment for data analysis [22] – and the front-end (client-side) was based on web design using HTML, CSS and Javascript via a web framework. Out of the two most popular Python-based web frameworks: Django and Flask, we chose Flask, due to its smaller size, robustness and extendibility [23]. We extended Flask with the Bootstrap library [24] allowing us to use pattern-based graphical elements, and with the Socket-IO library (Socket.IO) allowing us to use the Websockets protocol [26], which enable duplex server/client communication. To allow our backend to utilize the graphical capabilities of the web-browser, while giving the user at the front-end to benefits the familiarity of desktop-based applications, we used the Chromium Embedded Framework (CEF) [27] via the CEFPython package (CEFPython). CEF allow to embed Chromium-browser in desktop-based applications. We extended CEF the pywin32 package [29] to further enhance our control of the essential application properties (e.g. window size).

Our IDE uses many types of data entities including patient data (ID, age, education level etc.), group information (name, description, patient list etc.), task definition data (stimuli parameters) and task results (EEG data, signal strength, battery level, patient responses etc.). Here we used two type of databases: SQLite [30] and InfluxDB [31]. SQLite is a C/package-based local database, which can be programmed in Python via the sqlite3 package [32]. We used the SQLAlchemy [33], which includes Object-Relational Mapping (ORM) [34], to define an object-based database architecture. SQLAlchemy was embedded in our Flask-based front-end via the Flask-SQLAlchemy package [35]. InfluxDB is Time Series Database (TSDB) in which time-oriented data can be stored [36]. In contrast to other relational databases, TSDB allows for automated management of timestamps, efficient handing of continuous data streams, usage of influxQL queries which make time-based data-retrieval straightforward, and finally, it does not require a scheme (data template). The latter is particularly important here since different tasks generate different data entities and are coupled with different EEG apparatuses (e.g., different number/types of electrodes). We utilized the influxdb via the influxdb-python package [37].

Our framework utilized the ’Cortex API’ [38], which was developed by Emotive, to provide developers with access to their EEG product line. Data visualization was implemented in our IDE with the Plotly.js library [39]. We provide automated statistical analysis on task results, which we implemented using python numerical analysis package NumPy. Data can be exported to Excel for further analysis (implemented with the xlsxwriter package [40]). Finally, we used the Pyinstaller package [41] to provide an executable compiled environment.

## III. EEG Apparatus

One of the most accessible EEG lines of products is commercialized by Emotive. The Emotive EPOC was shown to have better accuracy then the common alternatives for control task [42], and it is capable of registering auditory event-related potentials (ERPs), which are comparable to state-of-the-art EEG system [43]. Our IDE currently supports the entire EMOTIV line of products, including the EPOC, Insight and Flex. The most widely utilized consumer-grade EEG device is the Emotiv EPOC headset [18]. The EPOC device has 14 Saline-soaked felt electrodes and two reference electrodes, placed according to the 10–10 international system of EEG electrode placement at AF3, F7, F3, FC5, T7, P7, O1, O2, P8, T8, FC6, F4, F8, and AF4, and references at P3, P4. The internal sampling rate of the device is 2048 Hz, down sampled to 128 Hz. Measurement resolution is 14 bits with 1 LSB $= 0.51\,\,\mu \text{V}$ (16-bit ADC, 2 bits instrumental noise floor discarded). Bandwidth range is 0.16 - 43 Hz with digital notch filters at 50 Hz and 60 Hz, and a dynamic range of 8400 $\mu \text{V}$(pp). Importantly, the EPOC has a 12 hours battery life, weighted only 1.2 kg. The Emotiv was not designed with ERP collection in mind, and therefore it has no hardware input for signals that allow recordings to be locked to a stimulus [44]. Data acquisition is therefore often modified to allow for research-driven EEG acquisition of distinct functional brain-states [21] and their underlying changes in specific EEG frequency bands under certain scalp electrodes (e.g., such as changes in alpha or theta oscillations during WM task-onset “active” state vs. WM task-offset “resting” state [45]). We note that our system is not unique to the Emotiv line of products and can be easily extended to support other EEG systems.

## IV. Neuropsychological Tasks

**FIGURE 3.**
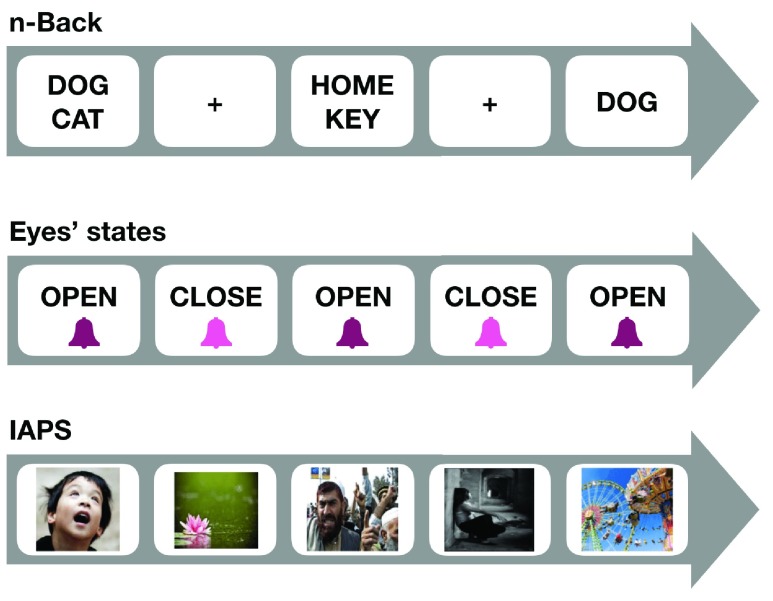
Supported types of neuropsychological tasks include: (top) N-back task, in which participants are sequentially exposed to a series of word-pairs at a fixed time interval, and required to match a single word to a word that appeared $n$-items ago; (middle) open vs close eyes task, in which patients are instructed, by verbal command, to open or close their eyes for fixed time intervals across fixed number of trials; and (bottom) affective/emotional processing task, in which participants indicate the emotional valence of images within the IAPS repository, by responding as quickly as possible as to whether it was unpleasant, neutral, or pleasant.

Our framework currently supports programming of three main types of neuropsychological tasks: N-back task (i.e. executive attention brain states), open vs close eyes task (i.e., resting brain states), and affective/emotional processing task (negative versus positive affect brain states) using the IAPS (**Figure 3**).

The $n$-Back task may represent a set of cognitive-neuropsychological operations, which were proposed by Kirchner and colleagues back in 1958 [46], aimed at assessing human short-term memory (later extended and modified to examine working memory). It was later used to reliably asses prefrontal cortex brain activity in humans [21]. During the current $n$-Back task version [21], participants are sequentially exposed to a series of word-pairs (potential targets) (e.g. DOG + CAT) at a fixed time interval. In an unexpected manner they are required to match a single word (probe) to a word that appeared $n$-items ago within potential targets (i.e. word-pairs). Participants are therefore required to continuously load and unload potential targets in working memory every two seconds. More so, in parallel, involved working memory (WM) neural mechanisms are constantly attempting to inhibit the activation of non-relevant items that appeared in previously encountered non-target displays. Therefore, this type of cognitive effort is likely to recruit executive attention mechanisms, which are conceptualized to endorse the capability to regulate goals in attempt to enable coherent and contextually appropriate behaviors (e.g., correct WM responses) in interference- rich conditions [21]. In our environment a researcher can flexibly program $n$-Back task parameters and compose the entire task. For example, the following task can be defined with just few clicks: a display of four types of trials ranging from n (1/3 of total trials) to n+3 (1/6 of total trials) stimuli displays that precede the probe onset, where half of the probes are matched, and the rest are not. Resting time (white screen) is defined every twelve trials. Here we used a similar task to find correlations between EEG readings and participant’s correct response time (reaction times), and their response accuracy (hits / correct rejections, total accuracy). Thus, our novel IDE, if validated across time and populations, will enable a non-invasive diagnostic user-friendly assessment of executive cognitive functioning in aging people and possibly in different clinical populations (e.g., pre-dementia patients).

During open/close eyes resting brain-states patients are instructed, by verbal command, to open or close their eyes for fixed time intervals across fixed number of trials. Shifting from open to closed eyes is triggered readings during “open” versus “closed” eyes brain-states with cognitive impairments in aging and Alzheimer’s disease populations [47], as well as in schizophrenia [48].

IAPS is an image repository that is used to elicit emotions and modulate attention in affective-behavioral neuroscience studies [49]. During the task participants indicate the emotional valence of each picture by responding as quickly as possible as to whether it was unpleasant, neutral, or pleasant. A recent study showed high identification rate (>85%) of women that experienced violence, using IAPS triggered EEG measurements [50]. In our environment we used an extended version of IAPS according to a valence-oriented study [49].

## V. Environment Description

**FIGURE 4.**
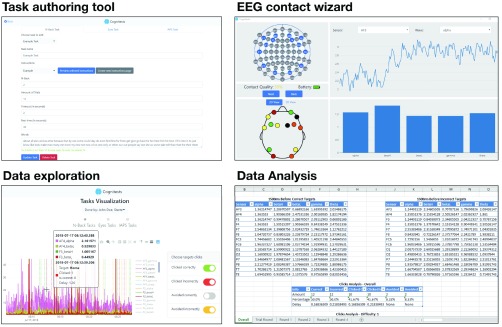
Chosen screenshots from the integrated development environment, representing a typical workflow with the system. The researcher defines the neuropsychological task using the task authoring tool, ensures good electrodes conductance using the EEG contact wizard, explore acquired data using the data exploration tool and export data statistical data using the data analysis tool.

Full interface description, as well as a detailed tutorial is given as Supplementary information. Briefly, the researcher can specify participants details (with full standard description), groups of participants and tasks. All can be saved or loaded from external files. Once a task is authored and chosen, the system searches for available headsets (via Bluetooth). When a headset is chosen, the EEG contact wizard is loaded, where the contact quality of the electrodes (2D / 3D) is monitored via a standard color scheme. In this wizard, band power from each electrode is recorded and displayed. Finally, a participant (or a group of participants) is chosen and the task is executed. Once data is acquired, data cleaned and analyzed to generate the EEG mean values represented in the results of different frequency bands. Data can be visualized with our data exploration wizard, in which each patient’s behavioral data can be viewed with his EEG data, clicks and colored indication lines for clicked correctly, incorrectly, and avoided. Finally, results can be exported to Excel. Few screen shots are shown in **Figure 4**.

## VI. Demonstration

A typical workflow with our system initiates with designing the neuropsychological task using our task authoring tool. Once the task is specified, the researcher defines the human subjects, individually, or from a previously defined group of subjects. Experiment starts with setting up the EEG equipment, connecting it wirelessly to the framework and then ensuring good electrode conductance using our EEG contact wizard. Once contact level is sufficient, the researcher chooses the desired task and execute it. The system stores the recorded data (EEG and responses (e.g. key presses)). The researcher can aggregate results from different groups, end explore the data using our tasks visualization tool. Finally, data and statistical analysis can be downloaded and explored using our data analysis tool. Full details are given in the supplemental manual.

**FIGURE 5.**
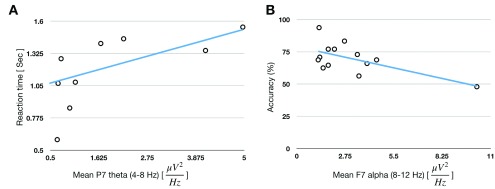
Framework demonstration. (A) Correlations were found between the participants’ mean reaction time (only for hits) and their mean theta (4-8 Hz) power under left parietal P7 electrode (Spearman’s rho = 0.56, $p =.044$) during WM “onset” brain state. (B) Correlation between performance (accuracy, percentage of correct responses) and mean alpha (8-12 Hz) power at left frontal F7 electrode (r =.65, p =.01) during resting periods at WM offset intervals.

We exemplified our framework by conducting a 2-Back task (recognizing target from two stimulus-displays ago, for further details see [21]) experiment (approved by Institutional Helsinki Ethics Committee, Herzog Medical Center, Jerusalem, Israel) with 14 elderly participants, aged between 66–83 years old. Participants signed an informed consent and were instructed to respond as fast as possible, by keypress, if they recognize a word-target that appeared within a pair of words from two displays ago. The task was approximately 15 minutes long, including instruction and one practice round of 7 trials. We investigated relationships between their behavioral results (accuracy and reaction time) and their EEG measurements (spectral density power of theta, alpha, beta and gamma frequency bands). We found significant correlations between the participants’ mean reaction time (only for hits) and their mean theta (4-8 Hz) power under left parietal P7 electrode (Spearman’s rho = 0.56, $p =.044$) during WM “onset” brain state (**Figure 5,A**) as well as between performance (accuracy, percentage of correct responses) and mean alpha (8-12 Hz) power at left frontal F7 electrode (r =.65, p =.01) during resting periods at WM offset intervals (**Figure 5,B**). This demonstration exhibits the capacity of our framework for task design, EEG interfacing and synchronization, data recording and analysis. More so, observed changes in EEG oscillations in the current study are supported by previous EEG-memory research [51], and indicated that WM task-related changes in frontal alpha at WM offset periods [45] and left parietal theta activity during WM onset periods are related to verbal WM performance in humans [52].

## VII. Discussion

One of the prominent commercial solution for conducting cognitive-neuropsychological research is E-Prime. While proved useful, E-Prime cost money and more importantly is closed for modifications and application-specific tailoring. Open-source software packages extended the available frameworks for cognitive-neuropsychological research with the development of PsyToolkit, which was recently shown to have comparable performance [53]. While open-source frameworks stand as an important milestone for lowering technical and financial barriers in EEG-driven research, this is often not enough. In their seminal paper: “End-User Development (EUD): An Emerging Paradigm” [54], Lieberman and colleagues emphasized user intuition as key to software design in an attempt to make software easy to use and develop. Examples for EUD range from spreadsheets to drag and drop webpages design. We designed our framework with EUD in mind - providing a powerful integrated environment for EEG-driven research.

Current available integrated EEG software framework include the OpenBMI [55], which was built upon MATLAB’s psychophysics toolbox [56] to provide experiment design and data analysis. This however required programming expertise, hardware interfacing and handling the overhead of participants’ data. On the other end, frameworks like Tatool [57], provide framework for experimental design with limited support for data analysis and hardware interfacing. Other framework such as PyGaze [58], CancellationTasks [59], or Sleep [60] provide application specific frameworks for eye-tracking, cancellation tasks and sleep data, respectively. Other frameworks such as the Neuroscience Information Toolbox [61] are focused on providing means for multimodal analysis. The development of EEG hardware also shows a similar trend, where expensive EEG equipment is being replaced by cheaper open sourced alternatives such as the Open Ephys+ EEG apparatus [62] and Creamino [63]. An important milestone for EEG-driven research is the transition from apparatus which are characterized with long electrodes placement time, scalp preparation and a wired cap connected to the computer (via EEG amplifier), to a relatively short long electrodes placement time, user-friendly wireless systems. The latter might open the door to new target populations. Further development of our framework may include an abstract layer which may provide support for other EEG systems via the separation of concerns principle. One such abstraction layer, was proposed by Cassani and colleagues, which developed a standardized hardware interface for EEG systems [64].

Our framework integrates the entire pipeline of cognitive-neuropsychological research, providing state-of-the-art graphical user interface for experiment design, data acquisition, data exploration and analysis. Thus, extending the range of packages available for cognitive neuroscientists, will allow researchers to concentrate on the creative part of their work instead of on time-consuming technical aspects that delay preparation and execution of EEG-driven clinical studies assessing human behavior and cognition.

## Appendix

Full description of the framework, a tutorial and installation instructions are given as an appendix to the manuscript.
